# Stiffening Potential of Lignocellulosic Fibers in Fully Biobased Composites: The Case of Abaca Strands, Spruce TMP Fibers, Recycled Fibers from ONP, and Barley TMP Fibers

**DOI:** 10.3390/polym13040619

**Published:** 2021-02-18

**Authors:** Ferran Serra-Parareda, Fabiola Vilaseca, Francesc X. Espinach, Pere Mutjé, Marc Delgado-Aguilar, Quim Tarrés

**Affiliations:** 1 LEPAMAP Research Group, University of Girona, Maria Aurèlia Capmany, 61, 17003 Girona, Spain; ferran.serrap@udg.edu (F.S.-P.); pere.mutje@udg.edu (P.M.); m.delgado@udg.edu (M.D.-A.); joaquimagusti.tarres@udg.edu (Q.T.); 2 Advanced Biomaterials and Nanotechnology, Department of Chemical Engineering, University of Girona, Maria Aurèlia Capmany, 61, 17003 Girona, Spain; fabiola.vilaseca@udg.edu; 3 Design, Development and Product Innovation, Department of Organization, Business, University of Girona, Maria Aurèlia Capmany, 61, 17003 Girona, Spain; 4 Chair on Sustainable Industrial Processes, University of Girona, Maria Aurèlia Capmany, 61, 17003 Girona, Spain

**Keywords:** natural fibers, biocomposites, stiffness, Young’s modulus, micromechanics

## Abstract

Biocomposites are composite materials where at least the matrix or the reinforcement phases are obtained from natural and renewable resources. Natural fibers for composite preparation can be obtained from annual plants, wood, recycled products, or agroforestry waste. The present work selected abaca strands, spruce fibers, recycled fibers from old newspaper, and barley fibers as raw materials to produce biocomposites, in combination with a biobased polyethylene. One very important feature in material science and for industrial applications is knowing how a material will deform under load, and this characteristic is represented by Young’s modulus. Therefore, in this work, the stiffness and deformation of the biocomposites were determined and evaluated using macromechanics and micromechanics analyses. Results were compared to those of conventional synthetic composites reinforced with glass fibers. From the micromechanics analysis, the intrinsic Young modulus of the reinforcements was obtained, as well as other micromechanics parameters such as the modulus efficiency and the length and orientation factors. Abaca strands accounted for the highest intrinsic modulus. One interesting outcome was that recycled fibers exhibited similar Young’s moduli to wood fibers. Finally, agroforestry waste demonstrated the lowest stiffening potential. The study explores the opportunity of using different natural fibers when specific properties or applications are desired.

## 1. Introduction

In recent years, the increasing environmental awareness in our society has led to the development of greener materials to displace fossil resources. In the sector of plastic composites, the use of natural fibers as a substitution for synthetic ones (i.e., glass, carbon, aramid) has gained interest both at the industrial and academic scales. Indeed, the applications of natural fiber-based composites are growing in many sectors such as automotive, building and construction, furniture, and packaging, among others [1,2]. This is due to the numerous benefits that natural fibers offer over synthetic fibers in terms of abundance, biodegradability, renewability, recyclability, low specific weight, and low cost while providing reasonable enhancement of mechanical properties [3,4,5]. Natural fibers for use in plastic-based composites can be obtained from different lignocellulosic resources, being (i) annual plants [6,7,8,9,10], (ii) wood [11,12,13,14,15], (iii) recycled products [16,17,18,19,20], and (iv) agroforestry waste [21,22,23,24]. The extraction of these natural fibers from the raw biomass may be achieved by different procedures, which mainly involve mechanical, semi-chemical, and chemical procedures [25,26,27]. Hence, depending on the raw lignocellulosic source and the fiber extraction method, the chemical and morphological composition of the fiber will vary accordingly, also affecting the final properties of the composite material. Therefore, understanding how the fiber typology and content can affect the properties of the composite is crucial when specific properties or applications are found [28,29,30]. 

However, the use of natural fibers as plastic reinforcement still does not solve all the environmental issues associated with the use of fossil resources, since polyolefin, followed by polyesters, still comprises the largest percentage of polymers employed in the composite sector [8,31]. To reduce the demand for fossil-based plastics, governmental regulations and companies’ social responsibility are boosting the development of a new generation of completely biobased polymers. Recent data, collected by European Bioplastics in cooperation with the research institute Nova-Institute, indicate that the global biobased polymer production is expected to increase from an approximate 0.93 million in 2019 to 1.09 million tons in 2024 [32].

Biobased polyethylene (BioPE) is a clear example of a polymer produced from a renewable feedstock, in this case, sugarcane mainly found in Brazil. BioPE was introduced in the market on a commercial scale in 2019, and it is predicted that by 2024, its production volume will increase by a ratio of six. The attractiveness of BioPE lies in its properties since the biopolymer is identical in its chemical, physical, and mechanical properties to fossil-based PE. Although at the beginning, the production of BioPE was considered unaffordable from an economic perspective in comparison to the conventional PE, recently, the cost of one barrel of ethanol obtained from sugarcane has become cost-competitive with one barrel of crude oil, making BioPE a viable option [33]. Furthermore, the incorporation of natural fibers is presented as an opportunity to improve the mechanical performance while reducing the material’s cost due to the low cost of natural fibers. Finally, it is possible to obtain a fully biobased material that can compete with conventional composites, giving rise to the so-called biocomposites.

The development of novel materials might fill demands that cannot be satisfied with existing materials. The viability of a material for a certain application is majorly determined by the dimensional stability, stiffness, and strength. In most of the applications, the stiffness requirements inflict greater limitations than other parameters do. This can be the case of applications where restrictions are imposed on allowable deflections [34]. The existence of these high demand requirements can only be satisfied by rigid materials. Thus, reinforcing fibers play a key role in providing the material the necessary stiffness to meet the industrial demands. In addition, the use of micromechanics models can be useful to reach a better understanding of the fibers’ stiffening mechanism within a polymer matrix, providing clear information on subjects such as the intrinsic stiffness of the fiber, the efficiency factor, and the influence of the length and the orientation on the final properties of the composites, among others. 

The stiffness of natural fiber-reinforced composites is mainly affected by the stiffness of the fibers and the matrix, fiber content, and its orientation inside the matrix. It has been reported that the quality at the fiber/matrix interphase has a minor influence on the composites’ stiffness, though the use of coupling agents to promote interfacial adhesion is largely recommended to increase the tensile strength and deformation capacity of the materials. The use of maleic anhydride polyethylene (MAPE) in polyethylene-based composites may increase matrix/fiber interactions and thus increase the interfacial adhesion. It is suggested that the anhydride groups from MAPE should form covalent bonds through esterification with fiber hydroxyl groups. On the other hand, MAPE’s PE chains diffuse into the unmodified PE chains from the matrix, forming a physical interaction (entanglement) [35,36,37,38,39,40].

The present work aims at exploring the stiffening potential of natural fibers in connection with all natural fiber resource possibilities. Additionally, the natural fibers employed in this study were mechanically extracted from the raw biomass, which means that its chemical composition has not been significantly affected (high-yield processes) and that the stiffening potential of each natural fiber may be trustfully compared. For this purpose, composites were produced using BioPE reinforced with abaca strands, spruce thermomechanical (TMP) fibers, recycled fibers from old newspaper (ONP), and barley TMP fibers. MAPE was added to the formulation as a coupling agent to enhance dispersion and interfacial adhesion. The composites with varying amounts of reinforcements were subjected to a mechanical test to evaluate their Young moduli. The study was carried out from macromechanics and micromechanics viewpoints. For comparison purposes, fully synthetic composites consisting of high-density polyethylene (HDPE) and glass fibers (GF) were prepared by the same approach, and their stiffness was evaluated.

## 2. Materials and Methods

### 2.1. Materials

Biocomposites were prepared using a biobased polyethylene (BioPE) as the polymer matrix, which was kindly supplied by Braskem (Sao Paulo, Brazil). The density of the polymer was 0.955 g/cm^3^, with a melt flow index of 20 g/10 min (190 °C; 2.16 kg). High-density polyethylene (HDPE) was used for the preparation of composite materials reinforced with glass fibers. HDPE was Rigidex HD5226EA and the density was 0.953 g/cm^3^, with a melt flow index of 26 g/10 min (190 °C; 2.16 kg). Polyethylene functionalized with maleic anhydride (MAPE) was used as the coupling agent. MAPE was Fusabond MB100D and was supplied by DuPont (Wilmington, DE, USA). 

Abaca strands were procured from Celesa (Tortosa, Spain) and used as received. Thermomechanical fibers from spruce were kindly provided by Norske Skog Saugbrugs (Halden, Norway) and used as received. Barley thermomechanical fibers were produced from barley straws, which were kindly provided by Mas Clarà S.A (Girona, Spain). More details about the obtention of barley thermomechanical fibers can be found in the literature [13]. The recycled fibers were obtained from old newspaper (ONP), which was kindly provided by Rotimpres S.L. (Punt Diari, Girona, Spain). According to the provider, the old newspaper contained 15 wt.% of calcium carbonate (CaCO_3_) and 85 wt.% of bleached thermomechanical fibers from birch, made by Stracel (Strasbourg, France). The obtention of recycled fibers is described in detail in previous works [16]. Glass fibers (GF) were produced by Vetrotex (Chambery Cedex, France) and supplied by Maben S.L. (Banyoles, Spain). Characteristics of GF are detailed in previous works [3].

Natural fibers were recovered from the composite materials by dissolving the polymer matrix in decahydronaphthalene (decalin) via Soxhlet extraction. The reagents used in the present work were provided by Sigma Aldrich and used as received.

### 2.2. Methods

**Figure 1:**
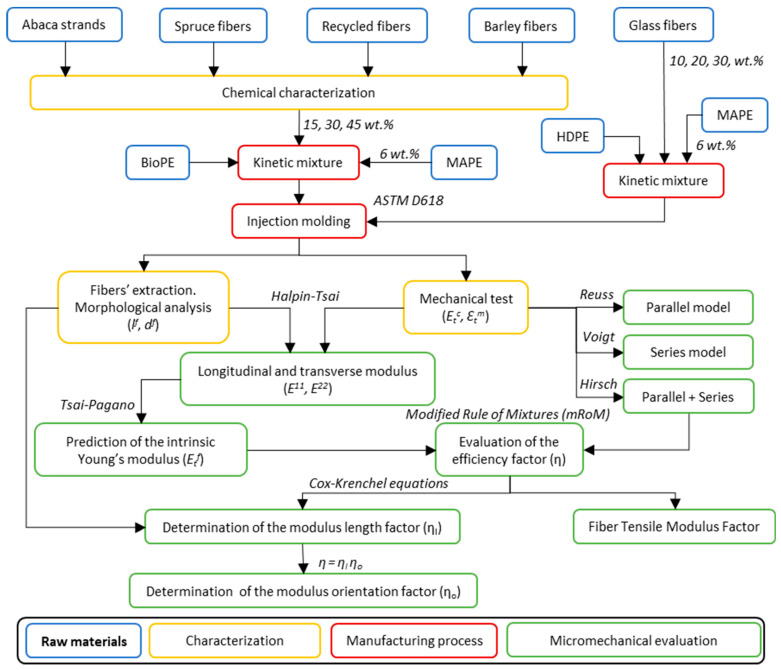
General workflow of the present investigation.

Figure 1 schematically presents the general workflow of this study, from raw materials and composites processing to characterization and micromechanics analysis.

#### 2.2.1. Chemical Characterization of the Fibers

The chemical constituents of the fibers were quantified following TAPPI standard protocols. First, fibers were subjected to an ethanol–toluene extraction to evaluate the context of extractives (TAPPI T264 om-07). Lignin was determined on the extractive-free samples following TAPPI T222 om-88. Parallelly, the ashes were measured following TAPPI T211 om-93. The holocellulose content was assumed from the difference between the total mass and the content of lignin, ashes, and extractives.

#### 2.2.2. Composites Processing

Biocomposites from BioPE and natural fibers were prepared at weight ratios of 15/85, 30/70, and 45/55 (fiber/matrix). Composite materials from HDPE and GF were formulated at weight ratios of 10/90, 20/80, and 30/70 (fiber/matrix). GF tended to aggregate at fiber contents over 30 wt.%, affecting the mechanical properties. Furthermore, the literature suggests the use of low GF contents to avoid damaging the equipment [41,42].

The matrix and reinforcement were mixed using a Gelimat kinetic mixer model G5S (Dusatec, Inc., Ramsey, NJ, USA). Initially, the oven-dried fibers were added at a rotor speed of 300 rpm. Then, the polymer and the coupling agent were incorporated maintaining the same speed. Afterward, the speed was gradually increased up to 3000 rpm and kept constant until the material finally melted and a homogeneous mix was achieved. The mixture was then discharged and cooled down at room temperature. The materials were palletized with a blade mill Retsch SM100 (Retsch Iberia, Llanera, Spain), equipped with a 5 mm mesh. The samples for the mechanical test were produced in an Aurburg 220 M 350-90U injection-molding machine (Aurburg, Loßburg, Germany) according to ASTM D3641.

#### 2.2.3. Mechanical Test

Before the mechanical testing, specimens were placed in a climatic chamber at 23 °C and 50% relative humidity, for 48 h. Young’s modulus values were acquired according to ASTM D618 using an InstronTM 1122 universal testing machine (Metrotec, Lezo, Spain), equipped with a 5 kN load cell. A gap between claws was set at 115 mm with a testing speed of 2 mm/min. For a more precise measurement of Young’s modulus, an MFA2 extensometer (Walter + Bai AG, Löhningen, Switzerland) was used.

#### 2.2.4. Fibers Extraction from the Composites

The morphological analysis was performed on the extracted fibers. Hence, the composites were subjected to Soxhlet extraction using decaline to dissolve the matrix. The process was completed after 48 h. Afterward, fibers were washed profusely with acetone to remove the impurities and suspended in water to avoid their aggregation.

The morphology was evaluated using a MORFI Compact Analyzer by Techpap SAS (Grenoble, France). Four analyses were carried out in each sample, each one measuring the morphology of 30,000 fibers. From the analysis, the mean fiber length and diameter were collected as the main important morphological parameters.

#### 2.2.5. Determination of Fiber Density and Volume Fractions

The application of the micromechanical models requires volume fractions instead of weight fractions. To this end, the density of the polymer and composite materials was measured using a pycnometer, with the density of the fibers being calculated from Equation (1):

(1) $$\rho^{f}=\frac{w^{f}\rho^{m}}{\frac{\rho^{m}}{\rho^{c}}\cdot \left(w^{m}+w^{f}\right)-w^{m}}$$

where $\rho^{c}$ and $\rho^{m}$ are the densities of the composite and matrix, respectively. The weight fractions of the matrix and the fiber are represented by $w^{m}$ and $w^{f}$, respectively. The fiber volume fraction ($V^{f}$) was then obtained from Equation (2), using the fibers’ density value.

(2) $$V^{f}=\frac{w^{f}/\rho^{f}}{w^{f}/\rho^{f}+w^{m}/\rho^{m}}$$

### 2.3. Micromechanics of Young’s Modulus

The intrinsic Young modulus of the reinforcing fibers was computed via the Hirsch model [43]. The model defines a linear combination between the parallel, Reuss, and serial, Voigt, models. The Reuss model defines a situation where each fiber is longitudinally aligned with the stress direction, whereas the fibers are transversally aligned with the stress direction in the Voigt model. The models have been illustrated in previous works [44]. The parallel, series, and Hirsch models are computed according to Equation (3), Equation (4), and Equation (5), respectively.

Parallel model:

(3) $$E_{t}^{c}=E_{t}^{f}\cdot V^{f}+E_{t}^{m}\left(1-V^{f}\right)$$

Series model:

(4) $$E_{t}^{c}=\frac{E_{t}^{f}\cdot E_{t}^{m}}{E_{t}^{m}\cdot V^{f}+E_{t}^{f}\cdot \left(1-V^{f}\right)}$$

Hirsch model:

(5) $$E_{t}^{c}=\beta \cdot \left(E_{t}^{f}\cdot V^{f}+E_{t}^{m}\left(1-V^{f}\right)\right)+\left(1-\beta \right)\frac{E_{t}^{f}\cdot E_{t}^{m}}{E_{t}^{m}\cdot V^{f}+E_{t}^{f}\cdot \left(1-V^{f}\right)}$$

where $E_{t}^{c}$ and $E_{t}^{m}$ are the experimental Young modulus of the composite and matrix, and $E_{t}^{f}$ is the intrinsic Young modulus of the fiber. Parameter β defines the stress transfer between the fiber and matrix and is principally influenced by the orientation of the fibers and by the stress concentration effects at the fiber ends. The literature agrees that β is around 0.4 in semi-aligned, short fiber composites processed using injection molding, though the present work studies how such parameters may change depending on the fiber typology and content [45,46].

The Tsai and Pagano model [47] and Halpin and Tsai [48] equations were also employed to predict the intrinsic Young modulus of fibers. Comparatively, Halpin and Tsai’s equations include morphological characteristics of the fibers, whereas the Hirsch model uses only experimental data from the mechanical test. Therefore, it is possible to evaluate the influence of the morphology on the intrinsic Young modulus of the fibers. The Tsai–Pagano model is defined as follows Equation (6):

(6) $$E_{t}^{C}=\frac{3}{8}E^{11}+\frac{5}{8}E^{22}$$

where $E^{11}$ and $E^{22}$ are the longitudinal and transverse moduli of the composite material. These factors may be determined following Halpin–Tsai equations, as shown in Equations (7) and (8):

(7) E11=1+2(lFdF)·ηlVF1−ηlVFEtm

(8) $$E^{22}=\frac{1+2\cdot \eta_{t}V^{F}}{1-\eta_{t}V^{F}}E_{t}^{m}$$

Parameters $\eta_{l}$ Equation (9) and $\eta_{t}$ Equation (10) are computed as:

(9) ηl=(EtFEtm)−1(EtFEtm)+2(lFdF)

(10) ηt=(EtFEtm)−1(EtFEtm)+2

Once the intrinsic Young modulus of the fibers has been computed either by the Hirsch or Tsai–Pagano model, it is possible to set a modulus efficiency factor that corrects the contribution of natural fibers to the stiffness of the material. The modulus efficiency factor (η) can be determined thanks to a modified rule of mixtures (mRoM) following Equation (11) [49]:

(11) $$E_{t}^{c}=\eta \cdot E_{t}^{f}\cdot V^{f}+E_{t}^{m}\cdot \left(1-V^{f}\right)$$

η is defined as the product between the modulus length factor ($\eta_{l}$) and modulus orientation factor ($\eta_{o}$), as observed in Equation (12). The parameters describe how the morphology and orientation of the fibers contribute to the stiffening efficiency thereof [18].

(12) $$\eta =\eta_{l}\cdot \eta_{o}$$

The modulus length factor can be calculated in agreement with Cox–Krenchel’s model Equations (13) and (14) [50,51]:

(13) ηl=1−tanh(β·lf2)(β·lf2)

(14) β=1rEtmEtf·(1−ν)·Lnπ4·Vf

where r and $l^{f}$ are the mean radius and diameter of the fibers, whereas ν defines the Poisson ratio of the matrix. The Poisson ratio (ν) of BioPE and HDPE is 0.45 [13]. By knowing the modulus efficiency factor and length factor, the modulus orientation factor is obtained from Equation (12). 

The neat contribution of fibers to the Young modulus of the composite may be evaluated by the fiber tensile modulus factor (FTMF) parameter. This parameter is obtained by rearranging the mRoM equation Equation (9). As a result, the contribution of the fibers to the stiffness of the composite, represented by $E_{t}^{c}-E_{t}^{m}\cdot \left(1-V^{f}\right)$, is presented as a function of the fiber volume fraction ($V^{f}$) at each fiber content. The slope of the line ($\eta \cdot E_{t}^{f}$) will define the contribution of the fibers to the Young modulus of the composite Equation (15). The parameter has been effectively used in the literature to define the stiffening capacity of natural fibers and is commonly used for comparison purposes within the same type of matrix.

(15) $$FTMF=\eta \cdot E_{t}^{f}=\frac{E_{t}^{c}-E_{t}^{m}\cdot \left(1-V^{f}\right)}{V^{f}}$$

## 3. Results

### 3.1. Chemical Composition of the Fibers

**Figure 2:**
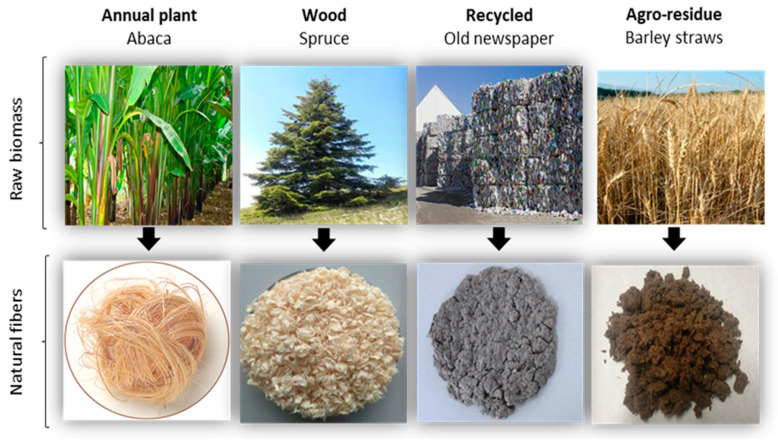
Illustrations of the different lignocellulosic resources and the mechanically extracted fibers.

Lignocellulosic materials with stiffening potential in plastic-based composites can be extracted from annual plants, wood, agroforestry waste, or recycled products. As representations of these categories, abaca strands, spruce fibers, barley fibers, and recycled fibers were selected and mixed with a biobased polyethylene (BioPE) matrix. For the readers’ convenience, Figure 2 presents illustrations of the raw biomass and the corresponding mechanically extracted fibers. The chemical composition of the fibers was analyzed as an important factor affecting the properties of the composite, and the results are reported in Table 1.

Table 1 reveals significant differences in the chemical composition of the fibers. Abaca strands owned the highest content of holocellulose at 87.2 wt.%, presenting lower quantities of lignin (8.8 wt.%). The holocellulose content decreased remarkably in fibers obtained from spruce, old newspaper, and barley, with values of 73.7, 73.2, and 77.7 wt.%, respectively. Instead, these samples contained higher amounts of lignin, with the content of lignin being higher in spruce fibers (25.8 wt.%) than in recycled (20.4 wt.%) and barley fibers (15.3 wt.%). Abaca and barley offered similar contents of extractives at 2.9 and 2.7 wt.%, respectively, whereas such component was lower for spruce and recycled fibers with values below 1 wt.%. Finally, the inorganic matter was elevated for recycled and barley fibers, whereas lower values were recorded for abaca and spruce.

The differences observed in the chemical composition of the fibers may be explained by the nature of the raw biomass, since the mechanical extraction of the fibers has a minor or even negligible effect on the chemical constituents. Non-woody plants such as abaca and barley usually present lower contents of lignin in comparison to woody ones, where the presence of lignin is considered crucial to ensure the maintenance of the fiber cell wall structure. Additionally, softwood species generally possess higher amounts of lignin compared to hardwood ones, which explains the higher content of lignin in spruce than in recycled fibers, which, according to the provider, has an elevated percentage of birch fibers [52]. Agroforestry waste usually contains higher amounts of extractives and ashes and less cellulosic materials, which usually makes such residues less attractive than other lignocellulosic resources. On the contrary, spruce wood has been typically characterized by its low content of extractives and ashes, along with other singularities that make spruce a commonly used raw material to produce thermomechanical fibers [53,54]. In the case of recycled fibers, the low content of extractives was expected due to the characteristics of birch wood, though the elevated content of ashes is attributed to the mineral fillers added during the production of recycled paper. 

As mentioned before, fibers extracted via entirely mechanical procedures are expected to preserve their chemical composition. However, the submission of the fibers to elevated temperatures before mechanical defibering, as in the case of barley and spruce, may slightly alter the chemical composition of the sample. Barley straws presented a content of lignin of 16.5 wt.%, whereas the extracted fibers showed a lignin content of 15.3 wt.%. For spruce fibers, the content of lignin went from 28.2 to 25.8 wt.%. Some extractives were also removed during the thermomechanical treatment since barley straws and spruce wood presented a content of extractives of 5.9 % and 7.1 wt.%, respectively. However, such differences are not considered relevant when it comes to the comparison between the different types of natural fibers.

### 3.2. Evaluation of Composites’ Stiffness

The fiber/matrix adhesion was enhanced by the addition of maleic anhydride polyethylene (MAPE). The amount of MAPE was optimized to reach the maximum tensile strength in the composites since it has been reported that Young’s modulus is not significantly affected by the strength of the fiber/matrix interphase [55]. It was found that 6 wt.% of MAPE concerning the fiber content yielded the higher increments in the tensile strength, causing an apparent improvement in the compatibility between the matrix and the fibers. Similar amounts of MAPE have been reported to enhance the fiber/matrix interfacial adhesion [56,57]. The coupling agent also favors the dispersion and distribution of the natural fibers inside the polymer matrix since both phases become more compatible. As a result, it is possible to prevent the formation of fiber aggregates and pores, which have negative effects on the material’s properties. Table 2 presents the density and volume fractions as well as the results from the mechanical test of both natural fiber and glass fiber composites, which were all formulated with 6 wt.% of MAPE with respect to the fiber content. 

Young’s moduli of biocomposites increased with the fiber content in all cases. This was, in part, expected due to the incorporation of a stiffer phase. Concurrently, the deformation at break tended to decrease due to the higher brittleness of the material. The incorporation of abaca strands yielded the highest increments in the stiffness of the biocomposites. This is explained both by the high content of holocellulose in abaca strands and the elevated aspect ratio (length/diameter) of the strands, as will be later observed in Table 3. Cellulose, computed inside the holocellulose content, is the major crystalline compound inside the fiber cell wall and provides the fiber strength and stiffness. Thereby, if the fiber is properly dispersed inside the composite, one can expect that the cellulose content of the fiber will contribute to the stiffness of the composite. Lignin also plays a major role in the effective stiffening capacity of the fibers. It was reported that optimal amounts of lignin at the kappa range of 40 to 50, corresponding to Klason lignin around 8 wt.%, favored the stress transfer capacity between phases and that lower contents of lignin would damage the fiber when the composite is subjected to a load [24]. Thus, the chemical composition of abaca strands provides the composite with high stiffness. 

Biocomposites from wood and recycled fibers exhibited similar Young’s moduli. This behavior is explained by the similarities in the chemical constituents of both materials since old newspapers are mainly composed of wood fibers. However, the deformation at break of composites containing recycled fibers dropped considerably in comparison to spruce fiber composites. It is known that the submission of recycled fibers to different recycling stages can cause irreversible damage to the fiber cell wall. Therefore, a more irregular and disorganized structure may be obtained. This affects the stress transfer between fibers when the composite is subjected to a load, apparently affecting the deformation capacity of the composite. 

**Figure 3:**
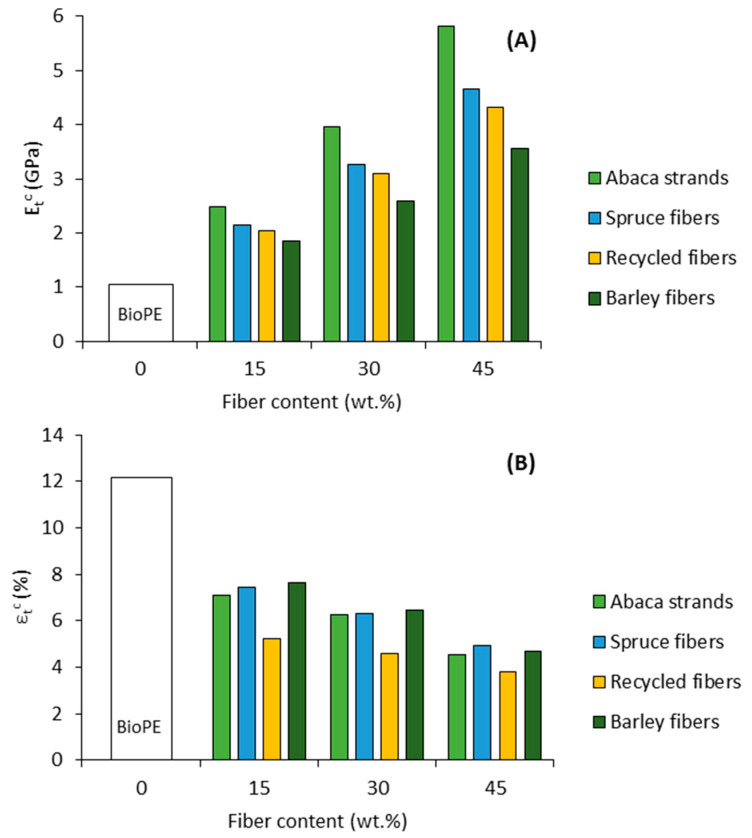
Evolution of Young’s modulus (**A**) and deformation at break (**B**) with the fiber content.

The incorporation of barley fibers to the composites yielded the lower increments in the Young modulus. However, the deformation in these materials was good, similar to that of abaca strands and spruce fibers. Advantageously, agroforestry waste provides clear environmental advantages, thanks to its abundance and opportunity to add value to a residue, which makes it an attractive source to produce biocomposites. Hence, the waste would suit low-cost material applications where the stiffness does not inflict major restrictions. The differences in Young’s modulus and deformation at break are better observed in Figure 3.

The stiffness of GF-based composites was higher than biocomposites, though the materials exhibit low deformation values, which results in difficulties to bear the applied load and limits their performance in some applications. Additionally, the poor interfacial adhesion between GF and polymers can result in poor tensile strengths and promote micro-cracks in different directions [58]. Moreover, natural fibers offer numerous advantages over GF, since they are not abrasive and do not damage the equipment, apart from being susceptible to further processing.

The density of biocomposites was considerably lower than GF-reinforced composites. This is due to the comparatively high density of GF, with a value of 2.45 g/cm^3^. The density of natural fibers was lower, with values ranging from 1 to 1.5 g/cm^3^, which contributed to the obtention of materials with a lower specific weight. This is considered as a major competitive advantage which can nowadays suit emerging needs among manufacturers of lightweight materials.

### 3.3. Contribution of Fibers to the Stiffness of Composites

**Figure 4:**
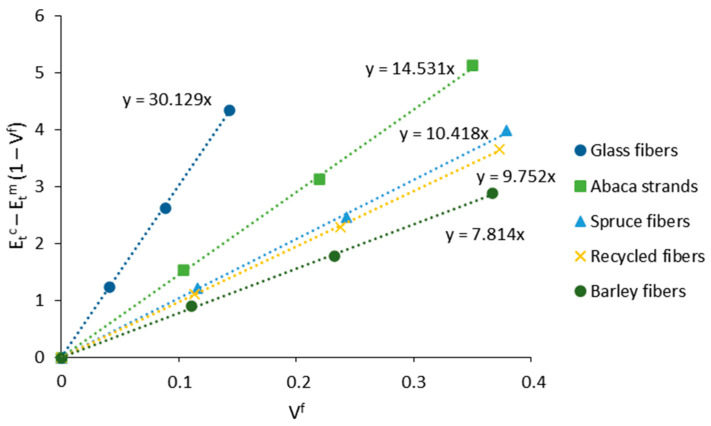
Contribution of the fibers to Young’s modulus of the composites via the fiber tensile modulus factor (FTMF).

The contribution of fibers to the stiffness of the materials was evaluated via a fiber tensile modulus factor Equation (15). The usefulness of this factor lies in its independence from the polymer matrix; thus, it can serve as a comparison with other fibers. Figure 4 presents the net contribution of the fibers against their volume fraction. The FTMF may be obtained from the slope of the line.

The FTMFs found were 30.1, 14.5, 10.4, 9.8, and 7.8 for GF, abaca strands, and spruce, recycled, and barley fibers, respectively. The FTMF of abaca strands was higher than other strands from annual plants such as hemp or jute, though still lower than flax or sisal [49,55,59]. Such differences are mainly attributed to the chemical composition and morphology of the fibers. For instance, flax and sisal possess higher amounts of cellulose, with minor amounts of lignin, extractives, and ashes [60]. 

The contribution of wood fibers was faintly higher than in the case of recycled ones. The values are in line with those reported in the literature for mechanical softwood fibers reinforced by other matrices, around 10.3. Between recycled and wood fibers, the literature shows very similar contributions to the stiffness of the composite. This contrasted with the present work, where the discrepancies were more notorious between the samples [3,61]. It is worth noting that the composition of recycled fibers is usually widespread and can vary depending on the raw materials, manufacturing process, and the desired properties of the final product. Accordingly, the FTMF is susceptible to change. The FTMF values in agroforestry waste also fluctuate depending on the initial source. In the present work, barley fibers showed lower contributions to the stiffness than other agroforestry wastes, for example, corn stover waste (8.74) [22], though similar values were detected in other types of waste [25].

Within the comparison of GF and natural fibers, it is observed that the net contribution of abaca at 45 wt.% was considerably higher than GF at 30 wt.%. At this point, the biocomposite showed improved deformation capacities and lower density, exhibiting the great potential of abaca and other annual plants to substitute GF. At 45 wt.% of spruce and barley, the neat contribution was slightly below the 30 wt.% of GF, whereas the contribution of 45 wt.% of barley fibers approximated the 20 wt.% of GF.

### 3.4. Micromechanics of Young’s Modulus

The intrinsic Young modulus of natural fibers is difficult to measure by direct testing. Alternatively, the use of micromechanics models has been widely accepted and recognized. Therefore, the intrinsic Young modulus of natural fibers was calculated via two different methodologies: (i) the Hirsch model and (ii) the Tsai–Pagano model using Halpin–Tsai equations. Comparatively, the Hirsch model uses solely experimental data from the tensile test, whereas Halpin–Tsai equations in the Tsai–Pagano model take into consideration the fibers’ morphology. Table 3 shows the mean fiber length and diameter along with the fibers’ intrinsic Young modulus. 

It is observed that the morphology of the fibers changed during the compounding processes since fibers suffered severe damage due to shear stress created during the mixing, eventually leading to fiber attrition [62]. This phenomenon was pronounced at higher fiber contents since the increment of the blend viscosity led to higher shear forces. This makes the evaluation of the fiber length and diameter after compounding crucial. From these parameters, the aspect ratio can be extracted (length/diameter) and incorporated into the Halpin–Tsai equations. 

The differences between the intrinsic Young moduli computed via the Hirsch and Tsai–Pagano models were not significant in spruce, recycled, and barley fibers. The good agreement between both models in these fibers indicates the usefulness of the Hirsch model with no requirement of morphological data. However, larger discrepancies between both models were found in abaca strands, which accounted for the higher aspect ratio. 

The highest intrinsic Young modulus was observed in abaca strands. Similar values are found in the bibliography in the case of strands from annual plants, such as alpha-grass (34.5 GPa), being lower in others such as hemp and jute strands [63,64]. The intrinsic stiffness decreased considerably for spruce and recycled fibers. According to the literature, wood fibers and recycled fibers exhibit similar intrinsic stiffness [34,65]. Young’s modulus was the lowest for agroforestry waste. Among such agricultural wastes, higher values are observed, for example, in the case of corn stover, rapeseed, orange tree, and others [22,46,66], manifesting the poor stiffness of barley fibers.

**Figure 5:**
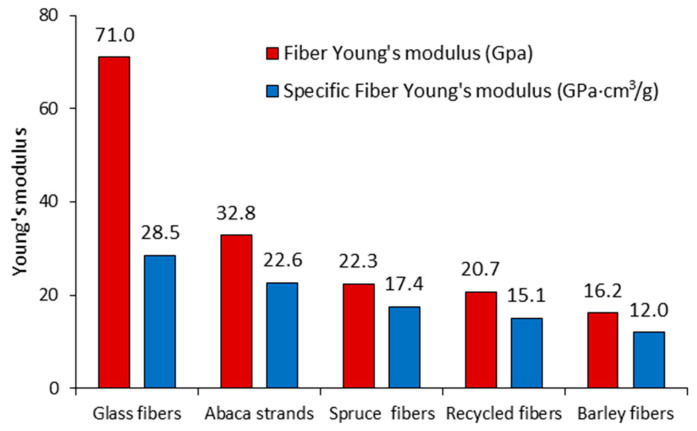
Comparison between the fiber Young moduli and fiber specific Young moduli.

The intrinsic Young modulus of GF was 71 GPa, which was found in line with the literature for GF [14]. Even though Young’s modulus of natural fibers is not as high as GF, the specific stiffness, defined as the ratio between Young’s modulus and the density of the fiber (E_t_^f^/ρ^f^), is comparable. This fact is given by the huge differences in the fibers’ density and makes natural fibers attractive in the composite field for specific lightweight material applications. The specific Young moduli of the fibers are shown in Figure 5.

**Figure 6:**
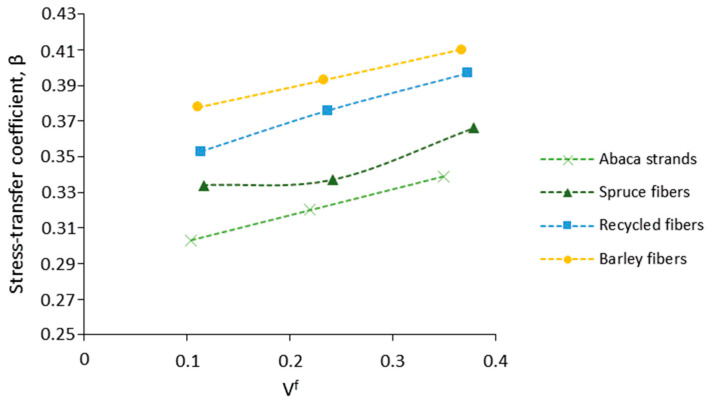
Evolution of beta coefficient with the fiber loading.

The utilization of the Hirsch model considers a stress transfer coefficient of β = 0.4, which has been proven to adequately suit the experimental and theoretical values in short, semi-aligned, fiber-reinforced composites. Note that the factor can vary between 0 and 1 and may be used to evaluate how the fiber loading affects the modulus of the composite in both the isostrain (β = 1) and isostress (β = 1) conditions. For instance, in randomly oriented fibers, β tends to a value of 0.1, whereas in semi-aligned fibers, the value tends to 0.4 [45]. As one may think, the factor may suffer variations depending on the orientation of the fibers inside the composite. Hence, the empirical factor β was corrected in composites reinforced by abaca, spruce, recycled, and barley fibers, in order to evaluate how the β parameter is affected by the fiber typology and fiber loading. For this purpose, the intrinsic fiber Young modulus given by the TP/HT model was incorporated into the Hirsch equation, and the corrected value of β was extracted. The results are shown in Figure 6.

The values of β tended to increase with the fiber volume fraction in all cases, as did the isostrain condition of the composites. An increment in the isostrain condition indicates that the composite is being more loaded parallel to the direction of the fibers, suggesting the alignment of the fibers with the loading direction. Therefore, the degree of anisotropy in the composites would increment with the fiber content. 

The modulus efficiency factor, extracted from the mRoM Equation (11), and the length factor, obtained from the Cox–Krenchel model Equations (13) and (14), were computed. Then, the orientation factor was isolated from Equation (12). The efficiency values were computed by using the intrinsic fiber Young modulus computed via the Hirsch and Tsai–Pagano models. The results are presented in Table 4.

The modulus efficiency factor was found inside the usual range for short, semi-aligned natural fibers, which is 0.4–0.56 [67]. Thereby, the value indicated that the composites took advantage of the stiffening capability of natural fibers, though with room for improvement. Comparatively, the factor decreased with the Tsai–Pagano values. This was normal since higher intrinsic Young’s moduli of natural fibers were found in comparison to the Hirsch model. In the case of GF, the modulus efficiency factor was 0.41.

The orientation factor is typically associated with compounding processes. Therefore, fibers’ semi-alignment is likely to occur during the injection molding. As observed, the highest alignment of the fibers was attained in barley composites, followed by recycled, spruce, and abaca, in that order. Additionally, the results indicate a progressive fiber alignment with the fiber content, which supports the increment of the isostrain condition observed in Figure 6. Sanomura and Kawamura studied the Young modulus of short fiber-reinforced thermoplastics with the orientation distribution assuming a rectangular distribution (square packing) of the fibers inside the matrix, as presented in Equation (16).

(16) $$\eta_{o}=\frac{\sin\alpha_{o}}{\alpha_{o}}\left(\frac{3-\nu }{4}\cdot \frac{\sin\alpha_{o}}{\alpha_{o}}+\frac{1+\nu }{4}\cdot \frac{\sin3\alpha_{o}}{3\alpha_{o}}\right)$$

where α_o_ denotes the fiber orientation limit angle. From the mean fiber orientation efficiency of the composites, a mean fiber orientation angle of 56°, 53°, 53°, and 51° was obtained for abaca, spruce, recycled, and barley composites, respectively, with 0° being a completely aligned scenario.

The length factor is characteristic of the fibers’ morphology and the attrition phenomena suffered by those during the blending of the phases. Consequently, the length factor increased with the aspect ratio of the reinforcing fibers, being higher for abaca strands. For spruce, recycled, and barley fibers, the length factors were not significantly different.

### 3.5. Evaluation of the Longitudinal and Transverse Moduli

**Figure 7:**
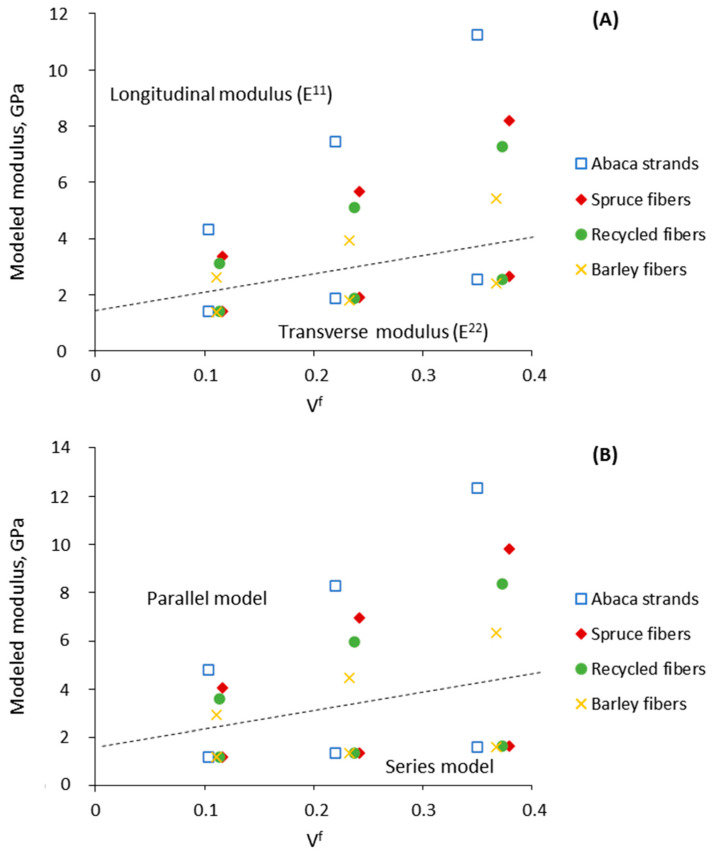
Modeled longitudinal and transverse Young’s modulus of biocomposites using Tsai–Pagano model and Halpin–Tsai equations (**A**). Modeled Young’s modulus via series and parallel models (**B**).

The level of anisotropy exhibited by the composites was analyzed. As explained, the Tsai–Pagano model creates a combination of the expected longitudinal (E^11^) and transverse (E^22^) moduli. Similarly, the Hirsch model incorporates the series and parallel models. As a result, the moduli in the longitudinal and transverse directions of the composites may be computed via the Tsai–Pagano and Hirsch models (Figure 7).

Figure 7 presents the values of the moduli in the longitudinal direction above the dashed line, whereas those in the transverse direction are observed below the line. The moduli in the longitudinal direction were always higher than in the transverse one, thus confirming the anisotropy of the composites. Such anisotropy should not be considered an inconvenience in certain applications where the stress happens in one direction, which can be the case of the building and construction sector. Noteworthily, similarities were perceived comparing the longitudinal modulus (E^11^) and the parallel model, as well as the transverse modulus (E^22^) and the series model. Note that the transverse modulus was not appreciably affected by the different types of fiber, which means that in the transverse direction, the modulus is not affected by the fiber typology, but rather the fiber content. 

Overall, a good understanding of the anisotropy of a material is of great interest when considering its potential in certain applications, since the stiffness can vary depending on the load direction and therefore the application of the composite.

## 4. Conclusions

In the present work, abaca strands, spruce fibers, old newspaper recycled fibers, and barley fibers were incorporated into a biobased polyethylene. Young’s modulus is seen as a useful property to predict the behavior of the material when subjected to a load, which is very important in construction, automotive, or other specific market sectors for composite materials. Therefore, the stiffness parameter was the focus of the study as a major influencing factor in determining the engineering use of natural fiber-based biocomposites. 

Abaca strand-reinforced composites accounted for the highest stiffness, followed by the values of wood fibers and recycled fibers, and, finally, barley fibers exhibited the lower stiffness values. In the case of wood and recycled fibers, mainly composed of original wood components, the little differences were attributed to the damage that occurred on recycled fibers during the recovery processes. The deformation capability of biocomposites was not significantly different, though it was observed that synthetic reinforcement such as GF fibers provided considerably lower strains than natural fibers, regarded as a competitive disadvantage.

The micromechanical analysis was of interest towards a better understanding of the performance of each type of natural fiber. From the computed values, the following remarks were pointed out: (i) the highest intrinsic modulus was attained for abaca strands, and the differences were mainly attributed to the chemical composition and morphology; (ii) small differences were observed between the application of the Hirsch and Tsai–Pagano models, indicating the usefulness of the first model with no requirement of morphological data; (iii) the modulus, length, and orientation factors were found within the range of natural fiber-reinforced composites; (iv) anisotropy was confirmed in biocomposites, and during the evaluation of composites’ anisotropy, similarities were found between the Tsai–Pagano model and the Hirsch model.

## Figures and Tables

**Table 1:** Chemical composition of abaca strands and spruce, recycled, and barley fibers.

	Abaca Strands	Spruce Fibers	Recycled Fibers	Barley Fibers
Holocellulose (wt.%)	87.3 ± 0.6	73.7 ± 0.8	73.2 ± 0.8	77.7 ± 0.6
Lignin (wt.%)	8.9 ± 0.2	25.8 ± 0.2	20.4 ± 0.4	15.3 ± 0.5
Extractives (wt.%)	2.9 ± 0.2	0.3 ± 0.3	0.8 ± 0.1	2.7 ± 0.1
Ashes (wt.%)	0.9 ± 0.1	0.2 ± 0.2	5.6 ± 0.3	4.3 ± 0.3

**Table 2:** Volume fractions (V^f^), density (ρ^c^), Young’s modulus (E_t_^c^), and deformation at break (ε_t_^c^) of natural fiber and glass fiber composites.

Matrix	Fiber Type	Fiber Content (wt.%)	ρ^c^ (g/cm^3^)	V^f^ (-)	E_t_^c^ (GPa)	ε_t_^c^ (%)
BioPE	-	-	0.96	-	1.06 ± 0.04	12.2 ± 0.2
	Abaca strands	15	1.01	0.104	2.49 ± 0.11	7.1 ± 0.2
		30	1.06	0.220	3.96 ± 0.09	6.3 ± 0.3
		45	1.13	0.350	5.81 ± 0.09	4.6 ± 0.2
	Spruce fibers	15	0.99	0.116	2.15 ± 0.12	7.5 ± 0.1
		30	1.03	0.242	3.26 ± 0.13	6.3 ± 0.2
		45	1.08	0.379	4.65 ± 0.07	4.9 ± 0.2
	Recycled fibers	15	1.04	0.114	2.05 ± 0.09	5.2 ± 0.2
		30	1.08	0.237	3.09 ± 0.05	4.6 ± 0.3
		45	1.14	0.373	4.32 ± 0.11	3.8 ± 0.1
	Barley fibers	15	1.00	0.111	1.85 ± 0.04	7.7 ± 0.2
		30	1.05	0.233	2.59 ± 0.09	6.5 ± 0.3
		45	1.10	0.367	3.55 ± 0.06	4.7 ± 0.2
HDPE	-	-	0.95	-	1.09 ± 0.03	11.1 ± 0.3
	Glass fibers	10	1.01	0.041	2.25 ± 0.08	5.5 ± 0.2
		20	1.09	0.089	3.67 ± 0.13	4.6 ± 0.1
		30	1.17	0.143	5.21 ± 0.14	3.2 ± 0.2

**Table 3:** Evaluation of the intrinsic Young modulus of natural fibers via the Hirsch model (E_t_^f^—Hirsch) and Tsai–Pagano model using Halpin–Tsai equations (E_t_^f^—TP/HT).

Natural Fiber	V^f^	l_w_^f^ (µm)	d^f^ (µm)	E_t_^f^—Hirsch (GPa)	E_t_^f^—TP/HT (GPa)	Discrepancy (GPa)
Abaca strands	0.104	1062 ± 32	22.3 ± 0.4	33.7	43.8	10.1
	0.220	966 ± 24	22.6 ± 0.2	32.1	39.5	7.4
	0.350	917 ± 17	22.4 ± 0.1	32.7	38.0	5.3
Spruce fibers	0.116	861 ± 26	25.2 ± 0.3	22.8	26.9	4.1
	0.242	792 ± 37	25.7 ± 0.3	21.8	25.4	3.6
	0.379	743 ± 33	25.4 ± 0.4	22.4	24.2	1.8
Recycled fibers	0.114	805 ± 41	21.4 ± 0.2	21.1	23.6	2.5
	0.237	746 ± 27	21.3 ± 0.3	20.5	21.7	1.2
	0.373	684 ± 26	21.4 ± 0.2	20.6	20.7	0.1
Barley fibers	0.111	596 ± 18	18.3 ± 0.1	17.2	18.1	0.9
	0.233	503 ± 26	18.5 ± 0.2	15.6	15.8	0.2
	0.367	387 ± 21	18.7 ± 0.2	15.8	15.4	-0.4

**Table 4:** Evaluation of the modulus efficiency factor (η), modulus length factor (η_l_), and modulus orientation factor (η_o_).

		Hirsch Model			TP/HT Model		
Natural Fiber	V^f^	η	η_l_	η_o_	η	η_l_	η_o_
Abaca strands	0.104	0.44	0.91	0.48	0.34	0.89	0.38
	0.220	0.44	0.92	0.48	0.36	0.92	0.39
	0.350	0.45	0.94	0.48	0.38	0.93	0.41
Spruce fibers	0.116	0.46	0.90	0.51	0.39	0.89	0.43
	0.242	0.47	0.92	0.51	0.40	0.91	0.44
	0.379	0.47	0.93	0.51	0.43	0.93	0.47
Recycled fibers	0.114	0.46	0.90	0.52	0.41	0.91	0.46
	0.237	0.47	0.91	0.51	0.44	0.93	0.48
	0.373	0.48	0.92	0.52	0.47	0.94	0.50
Barley fibers	0.111	0.48	0.88	0.54	0.45	0.91	0.50
	0.233	0.49	0.90	0.54	0.48	0.92	0.53
	0.367	0.49	0.90	0.55	0.51	0.92	0.55

## Data Availability

The processed data required to reproduce these findings cannot be shared at this time as the date also forms part of an ongoing study.
